# Increased circulating desmosine and age-dependent elastinolysis in idiopathic pulmonary fibrosis

**DOI:** 10.1186/s12931-018-0747-6

**Published:** 2018-03-20

**Authors:** Bart de Brouwer, Marjolein Drent, Jody M. W. van den Ouweland, Petal A. Wijnen, Coline H. M. van Moorsel, Otto Bekers, Jan C. Grutters, Eric S. White, Rob Janssen

**Affiliations:** 1 Department of Pulmonary Medicine, Canisius-Wilhelmina Hospital, Weg door Jonkerbos 100, 6532 SZ Nijmegen, The Netherlands; 2 Center of Interstitial Lung Diseases, Department of Pulmonology, St. Antonius Hospital, Nieuwegein, The Netherlands; 3 Department of Pharmacology and Toxicology, FHML, Maastricht University, Maastricht, The Netherlands; 4 Department of Clinical Chemistry, Canisius-Wilhelmina Hospital, Nijmegen, The Netherlands; 5 Department of Clinical Chemistry, Central Diagnostic Laboratory, Maastricht University Medical Center+, Maastricht, The Netherlands; 6 Division of Pulmonary and Critical Care Medicine, University of Michigan Medical School, Ann Arbor, Michigan USA

## Abstract

Although chronic obstructive pulmonary disease (COPD) and idiopathic pulmonary fibrosis (IPF) seem to be opposite entities from a clinical perspective, common initial pathogenic steps have been suggested in both lung diseases. Emphysema is caused by an elastase/anti-elastase imbalance leading to accelerated elastin degradation. Elastinolysis is however, also accelerated in the IPF patients’ lungs. The amino acids desmosine and isodesmosine (DES) are unique to elastin. During the degradation process, elastases liberate DES from elastin fibers. Blood DES levels consequently reflect the rate of systemic elastinolysis and are increased in COPD. This is the first report describing elevated DES levels in IPF patients. We also demonstrated that the age-related increment of DES concentrations is enhanced in IPF. Our current study suggests that elastinolysis is a shared pathogenic step in both COPD and IPF. Further investigation is required to establish the relevance of accelerated elastin degradation in IPF and to determine whether decelerating this process leads to slower progression of lung fibrosis and better survival for patients with IPF.

## To the Editor,

Although chronic obstructive pulmonary disease (COPD) and idiopathic pulmonary fibrosis (IPF) seem to be opposite entities from a clinical perspective, they often coexist in the same patient [1], and shared pathogenic mechanisms have been suggested between these two lung diseases [2]. It is generally thought that emphysema is caused by an elastase/anti-elastase imbalance leading to accelerated elastin degradation. Elastinolysis, however, is also accelerated in the lungs of patients with IPF [3]. Similarly, matrix metalloproteinases are elastases that play a pivotal role in COPD but are also implicated in the pathogenesis of IPF [4].

The elastin precursor tropoelastin is mainly produced perinatally [5]. Tropoelastin monomers are subsequently aligned, and the resulting tropoelastin polymers are crosslinked into durable elastin fibers by the enzyme lysyl oxidase [5]. This crosslinking process gives rise to the amino acids desmosine and isodesmosine (DES), which are unique to mature elastin [5]. During the elastin degradation process, elastases liberate DES from elastin fibers [6]. Consequently, plasma (p)DES levels reflect the rate of elastinolysis. pDES levels are increased in COPD patients [7], and DES concentrations are also elevated in bronchoalveolar lavage fluid of patients with IPF [3]. However, data regarding pDES in IPF are lacking. Huang *et al*. recently demonstrated that elastin degradation accelerates during ageing in subjects with or without obstructive lung disease, but that this age-related DES increment is amplified in patients with COPD [8]. Whether this also holds true for patients with IPF is currently unknown.

By analogy to COPD, we hypothesized that pDES levels would be elevated and that the age-related pDES increment would be enhanced in IPF patients compared to controls with no lung disease.

pDES levels were measured by liquid chromatography tandem-mass spectrometry in 154 IPF patients from Maastricht (59 subjects, 30 males, 59±11 years, 11 active smokers, 44 never smokers, 4 former smokers), from Nieuwegein (20 subjects, 18 males, 66±9 years, 16 former smokers, 4 never smokers) and from Ann Arbor (75 subjects, 56 males, 67±7 years, 38 former smokers, 37 never smokers) as previously described [8, 9]. IPF was diagnosed by a multidisciplinary team of experts according to international guidelines [10]. For each pDES measurement in an IPF patient, a virtual age-matched pDES value was calculated using published pDES equations: non-smoking controls (50+2.91*age ng/L), smoking controls (70+3.12*age ng/L) and COPD patients (50+6.57*age ng/L) [8]. pDES levels were also measured in 142 patients with moderate to severe COPD (121 males; 68±9 years) and 84 controls (29 males; 46±13 years). Correlations between forced vital capacity (FVC) and pDES values were investigated. Analysis of covariance (SPSS version 24) was used to compare pDES levels between groups, adjusted for age. Data are presented as estimated marginal means ± standard error of the mean.

**Fig. 1:**
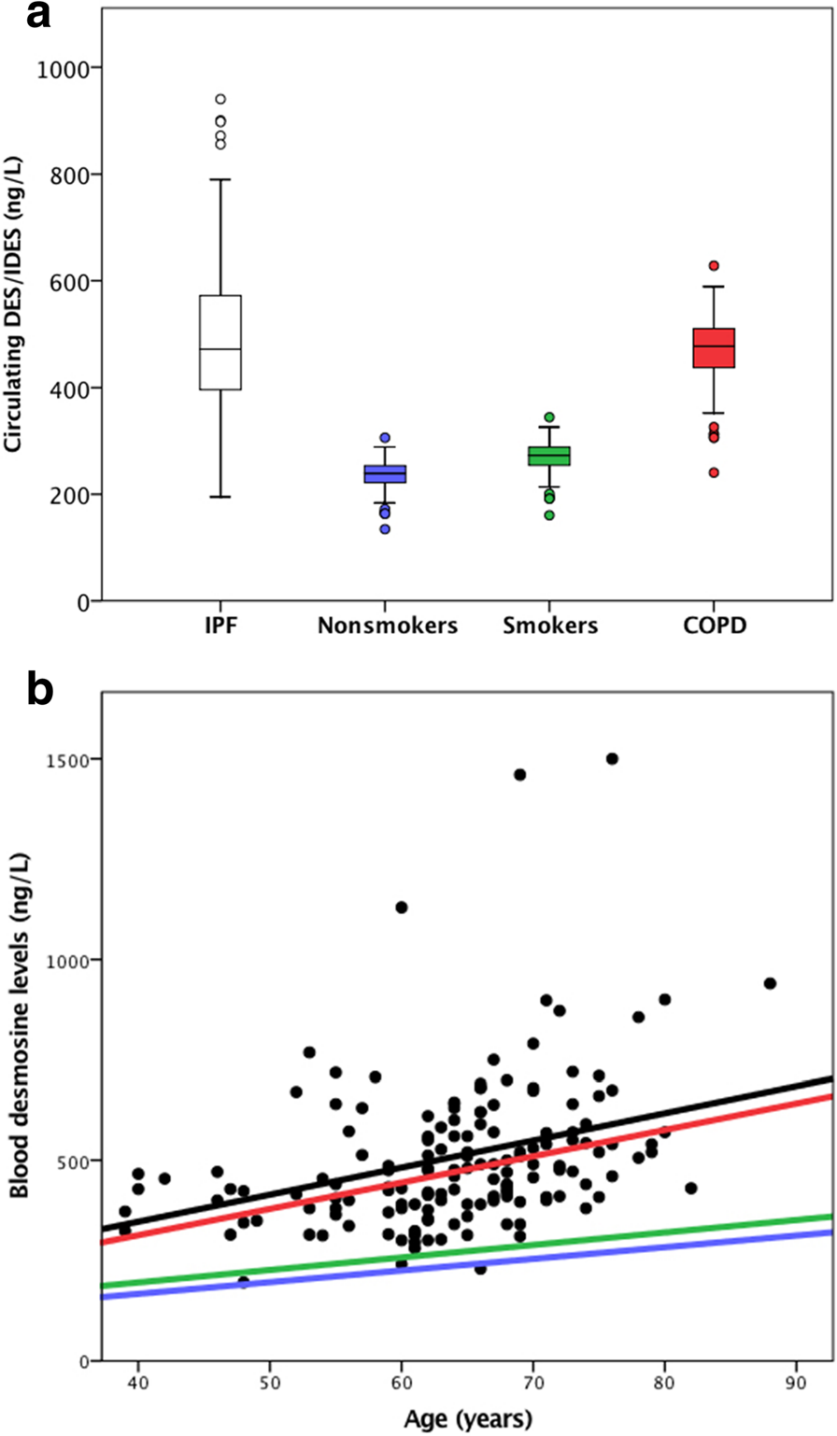
Circulating desmosine levels in IPF patients, based on measurements, and in three other groups, based on virtual age-matched controls calculated with Huang *et al.*’s equations: non-smoking controls (50 + 2.91*age ng/L; blue), smoking controls (70 + 3.12*age ng/L; green) and COPD patients (50 + 6.57*age ng/L; red) [8]. **a**) Boxplots showing blood desmosine levels (5^th^ percentile, 1^st^ quartile, median, 3^rd^ quartile, and 95^th^ percentile). Significantly higher blood desmosine levels were found in IPF patients compared to smoking or non-smoking controls and COPD patients. **b**) Scatterplot in which the black dots represent circulating desmosine measurements and the black line the deduced equation line in IPF patients (76 + 6.76*age ng/L).

A significant correlation was found between age and pDES (p<0.0005). The equation for the pDES regression line in IPF patients was 76 (95% CI -117–270)+6.76 (95% CI 3.75–9.77)*age (ng/L). Significantly higher pDES levels were found in IPF patients (507±8 ng/L) compared to age-adjusted reference values of non-smoking controls (236±8 ng/L, p<0.0005), of smoking controls (269±8 ng/L, p<0.0005) and of COPD patients (469±8 ng/L, p=0.001; Fig. 1a and 1b) [8]. No significant differences in pDES levels were found between the three IPF cohorts (p=0.785). pDES levels were 509±24 ng/L in IPF patients from Maastricht, 481±40 ng/L in IPF patients from Nieuwegein, and 512±21 ng/L in IPF patients from Ann Arbor. Significantly higher pDES levels were found in both COPD (457±23 ng/L, p=0.006) and IPF patients (*p*<0.0005) compared to controls (337±32 ng/L). No significant differences in pDES levels were found between IPF and COPD patients (p=0.2). FVC data at baseline were available from the Nieuwegein and Ann Arbor cohort; no significant correlation was found between pDES levels and %FVC of predicted at baseline (*p*=0.3). FVC follow-up data were available from the Nieuwegein cohort; a significant correlation was demonstrated between pDES levels and the change in %FVC of predicted per year (*p*=0.02).

We have demonstrated elevated pDES levels in IPF patients from three independent cohorts compared to smoking and non-smoking controls and COPD patients. We also found amplified age-related DES increment in IPF similar to what was previously described for COPD [8]. We also demonstrated a correlation of pDES with lung function decline.

The strong effect of age on pDES levels in IPF patients once again stresses the importance of taking age into account as a covariate when interpreting concentrations of this biomarker. It has previously been noted that pDES increases both in controls and COPD patients during ageing, but that the gain factor due to age is much higher in the latter [8]. We now demonstrate that age-related pDES increment is also amplified in IPF, which corroborates to the acceleration of ageing processes observed in patients with IPF [11].

It has previously been demonstrated that pDES levels are related to emphysema progression in COPD patients with alpha-1 antitrypsin (AAT) [12]. Based particularly on the observation that emphysema is often present in lungs of IPF patients [1], we suspect there are similarities in the pathogenesis of lung fibrosis and emphysema. Our present results of accelerated elastin degradation in IPF patients –with pDES concentrations at least as high as previously described in COPD– may be interpreted as circumstantial evidence that elastinolysis could be a shared pathogenic step between COPD and IPF. Interestingly, data from an animal model have suggested a decisive role for elastin metabolism in the divergence between lung fibrosis and emphysema during the pathological sequelae of lung injury [2]. Whereas intratracheal administration of cadmium chloride caused fibrosis in hamsters’ lungs, combining this toxin with an inhibitor of the elastin crosslinker lysyl oxidase led to emphysema [2]. Additional studies are needed to unravel which specific steps of elastin metabolism are involved in IPF pathogenesis.

Previous research has mainly focussed on pDES as a biomarker of systemic elastin degradation in COPD [13]. Our present findings of even higher pDES in IPF patients than in patients with COPD also justifies exploring the role of this biomarker in IPF. When interpreting pDES levels in patients with COPD, it is important to realize that elastin degradation in COPD is not only accelerated in the lungs but also in other dynamic tissues such as arteries and skin [14]. In contrast to the severity and progression of emphysema, the burden of coronary artery calcification (CAC) has been associated with pDES levels in COPD patients with normal AAT levels [7], which might indicate that arteries are the main contributors to pDES in this patient group. CAC is also prevalent in IPF patients [1], and future studies are therefore needed to assess whether pDES also relates to CAC in IPF. The pulmonary arteries may also be a potential source of circulating elastin fragments in IPF patients, given that pDES levels are elevated in patients with pulmonary arterial hypertension and that pulmonary hypertension is another common comorbidity of IPF [1, 15]. Furthermore, DES seems to be a predictor of mortality in COPD and cystic fibrosis [7, 16]. It is therefore also relevant to assess the prognostic value of pDES for survival in patients with IPF.

We regard deceleration of elastin degradation as a potential attractive novel therapeutic target in COPD. This intriguing new concept was illustrated by a recently published *post hoc* study in which AAT augmentation therapy resulted in a decrease of pDES levels in AAT deficient COPD patients [12]. In IPF, the treatment landscape has shown positive changes following the introduction of the two anti-fibrotic drugs pirfenidone and nintedanib [10]. Although the complex and pleiotropic properties of these agents have not been precisely unravelled, their mechanisms of action seem to be quite different. Whether inhibition of elastin catabolism is a relevant therapeutic effect of these anti-fibrotics is currently unknown. Additional studies are needed to assess whether pirfenidone and/or nintedanib have effects on pDES levels. Furthermore, it is important to study whether modifications in the rate of elastin degradation run parallel to changes in the disease course of patients with IPF. In other words, whether decelerating and accelerating elastin degradation in IPF translates into slower and faster disease progression, respectively. This may be suggested by the association we found between baseline pDES levels and FVC decline. We might speculate about validating combinations of IPF biomarkers to make a more educated choice between pirfenidone and nintedanib in individual patients, based on expected efficacy rather than adverse side effects, as is the current practice [10]. Future studies have to reveal whether there may be a role for pDES in such a panel.

In summary, this is the first study describing elevated pDES levels in IPF patients, which were even higher than in COPD patients. We also demonstrated that the age-related increment of pDES concentrations is enhanced in IPF. Additional studies are needed to establish the relevance of accelerated elastin degradation in IPF and to determine whether decelerating this process translates into slower progression of lung fibrosis and better survival for patients with IPF.

## Abbreviations

AAT: Alpha-1 antitrypsin
CAC: Coronary artery calcification
COPD: Chronic obstructive pulmonary disease
DES: Desmosine and isodesmosine
FVC: Forced vital capacity
IPF: Idiopathic pulmonary fibrosis
